# *Bartonella* spp. Bacteremia and Rheumatic Symptoms in Patients from Lyme Disease–endemic Region

**DOI:** 10.3201/eid1805.111366

**Published:** 2012-05

**Authors:** Ricardo G. Maggi, B. Robert Mozayeni, Elizabeth L. Pultorak, Barbara C. Hegarty, Julie M. Bradley, Maria Correa, Edward B. Breitschwerdt

**Affiliations:** North Carolina State University, Raleigh, North Carolina, USA (R.G. Maggi, E.L. Pultorak, B.C. Hegarty, J.M. Bradley, M. Correa, E.B. Breitschwerdt);; Translational Medicine Group, PC, North Bethesda, Maryland, USA (B.R. Mozayeni)

**Keywords:** Bartonella, bacteremia, blood, arthritis, myalgia, PCR, DNA sequencing, bacteria, Lyme disease, rheumatic, Lyme disease

## Abstract

*Bartonella* spp. infection has been reported in association with an expanding spectrum of symptoms and lesions. Among 296 patients examined by a rheumatologist, prevalence of antibodies against *Bartonella henselae, B. koehlerae,* or *B. vinsonii* subsp. *berkhoffii* (185 [62%]) and *Bartonella* spp. bacteremia (122 [41.1%]) was high. Conditions diagnosed before referral included Lyme disease (46.6%), arthralgia/arthritis (20.6%), chronic fatigue (19.6%), and fibromyalgia (6.1%). *B. henselae* bacteremia was significantly associated with prior referral to a neurologist, most often for blurred vision, subcortical neurologic deficits, or numbness in the extremities, whereas *B. koehlerae* bacteremia was associated with examination by an infectious disease physician. This cross-sectional study cannot establish a causal link between *Bartonella* spp. infection and the high frequency of neurologic symptoms, myalgia, joint pain, or progressive arthropathy in this population; however, the contribution of *Bartonella* spp. infection, if any, to these symptoms should be systematically investigated.

## Abstract 2

Prevalence of *Bartonella* spp. was high, especially among patients with a history of Lyme disease.

The genus *Bartonella* comprises at least 26 species or subspecies of vector-transmitted bacteria, each of which has evolved to cause chronic bacteremia in >1 mammalian reservoir hosts (*1**–**4*). Among these, bartonellae of 14 species or subspecies have been implicated in zoonotic diseases (*5**,**6*), including cat-scratch disease, which is caused by *B. henselae* transmission during a cat bite or scratch and characterized by acute onset of self-limiting fever and regional lymphadenopathy (*7**–**9*). Recent observations, however, are causing a paradigm shift from the assumption that infection with a *Bartonella* sp. consistently induces an acute, self-limiting illness to the realization that subsets of infected, immunocompetent patients can become chronically bacteremic (*10**–**15*).

After *B. henselae* was confirmed as the primary cause of cat-scratch disease in the early 1990s, several reports described an association between the newly identified bacterium and rheumatic disease manifestations, variously described as rheumatoid, reactive, or chronic progressive polyarthritis (*16**–**20*). One study, however, failed to isolate *B. henselae* from synovial fluid of 20 patients with chronic arthritis (*21*). Because epidemiologic evidence supports an association between rheumatic symptoms and cat-scratch disease and because arthritis is a primary disease manifestation of *Borellia burgdorferi* infection (Lyme disease), we explored whether antibodies against and bacteremia with *Bartonella* spp. can be detected in patients examined for arthropathy or chronic myalgia. Our primary objective was to determine the serologic and molecular prevalence of *Bartonella* spp. bacteremia in patients referred to a clinical rheumatologist. We also compared self-reported symptoms, health history, and demographic factors with *Bartonella* spp. bacteremia as determined by an enrichment blood culture platform combined with PCR amplification and DNA sequencing, when possible, to determine the *Bartonella* species and strain. This study was conducted in conjunction with North Carolina State University Institutional Review Board approval (IRB# 164–08–05).

## Materials and Methods

### Study Population

For this cross-sectional study, we enrolled only patients examined by a rheumatologist in the Maryland–Washington, DC, USA, area from August 25, 2008, through April 1, 2009. Because *Bartonella* spp. are known to primarily infect cells within the vascular system, including erythrocytes, endothelial cells, and potentially circulating and tissue macrophages (*1**,**5**,**6*), selection was biased by patients who had historical, physical examination, or laboratory evidence of small vessel disease, including a subset of patients with a prior diagnosis of Lyme disease or chronic post–Lyme syndrome. We also included patients with chronic joint pain, prior documentation of synovial vascular inflammation, or a diagnosis of rheumatoid arthritis.

A standardized 5-page questionnaire was mailed to each participant for self-report. The questionnaire collected information about demographics, animal/arthropod exposure, history of visiting a medical specialist, outdoor activity, self-reported clinical symptoms, and concurrent conditions. Questionnaires were returned to the Intracelluar Pathogens Research Laboratory at North Carolina State University, College of Veterinary Medicine, Raleigh, North Carolina, USA, where results were entered into an electronic database.

### Sample Collection

From each patient, the attending rheumatologist aseptically obtained anticoagulated blood samples (in EDTA tubes) and serum samples and shipped them overnight to the laboratory. Patient variations included timing of sample collection relative to onset of illness, duration of illness, current illness severity, and prior or recent use of antimicrobial drugs. The samples were then processed in a limited-access laboratory.

### Sample Processing

#### Immunofluorescence Antibody Assay

To determine the antibody titer to each *Bartonella* species or subspecies, we used *B. henselae*, *B. koehlerae*, and *B. vinsonii* subsp. *berkhoffii* (genotypes I, II, and III) antigens in a traditional immunofluorescence antibody (IFA) assay with fluorescein conjugated goat anti-human IgG (Pierce Antibody; Thermo Fisher Scientific, Rockford, IL, USA) (*10**,**12**,**22*). To obtain intracellular whole bacterial antigens for IFA testing, we passed isolates of *B. henselae* (strain Houston-1, ATCC #49882); *B. koehlerae* (NCSU FO-1–09); and *B. vinsonii* subsp. *berkhoffii* genotypes I (NCSU isolate 93-CO-1, ATCC #51672), II (NCSU isolate 95-CO-2), and III (NCSU isolate 06-CO1) from agar-grown cultures into *Bartonella*-permissive tissue culture cell lines: AAE12 (an embryonic *Amblyomma americanum* tick cell line) for *B. henselae*, DH82 (a canine monocytoid cell line) for *B. koehlerae*, and Vero (a mammalian fibroblast cell line) for the *B. vinsonii* genotypes*.* Heavily infected cell cultures were spotted onto 30-well Teflon coated slides (Cel-Line; Thermo Fisher Scientific), air dried, acetone fixed, frozen, and stored. Serum samples were diluted in a phosphate-buffered saline solution containing normal goat serum, Tween-20, and powdered nonfat dry milk to block nonspecific antigen binding sites and then incubated on antigen slides. All available patient serum was screened at dilutions from 1:16 to 1:64. Samples reactive at a 1:64 dilution were further tested with 2-fold dilutions to 1:8192. As in previous studies, we defined a seroreactive antibody response against a specific *Bartonella* sp. antigen as a threshold titer of 64 (*10**–**15**,**23**,**24*).

#### *Bartonella* α Proteobacteria Growth Medium Enrichment Culture

Each sample was tested by PCR amplification of *Bartonella* spp. DNA before and after enrichment of blood and serum in *Bartonella* α Proteobacteria growth medium (BAPGM) (*10**–**14**,**23**–**26*). The BAPGM platform incorporates 4 PCR steps, representing independent components of the testing process for each sample, as follows: step 1) PCR amplifications of *Bartonella* spp. after DNA extraction from whole blood and serum; steps 2 and 3) PCR after whole blood culture in BAPGM for 7 and 14 days; and step 4) PCR of DNA extracted from subculture isolates (if obtained after subinoculation from the BAPGM flask at 7 and 14 days onto plates containing trypticase soy agar with 10% sheep whole blood, which are incubated for 4 weeks). To avoid DNA carryover, we performed PCR sample preparation, DNA extraction, and PCR amplification and analysis in 3 separate rooms with a unidirectional work flow. All samples were processed in a biosafety cabinet with HEPA (high-efficiency particulate air) filtration in a limited-access laboratory.

Methods used to amplify *Bartonella* DNA from blood, serum, and BAPGM liquid culture and subculture samples included conventional PCR with *Bartonella* genus primers targeting the 16S-23S intergenic spacer region (ITS) and a second PCR with *B. koehlerae* ITS species-specific primers, as described (*13**,**25**–**29*). Amplification of the *B. koehlerae* ITS region was performed by using oligonucleotides Bkoehl-1s: 5′-CTT CTA AAA TAT CGC TTC TAA AAA TTG GCA TGC-3′ and Bkoehl1125as: 5′-GCC TTT TTT GGT GAC AAG CAC TTT TCT TAA G-3′ as forward and reverse primers, respectively. Amplification was performed in a 25-µL final volume reaction containing 12.5 µL of Tak-Ex Premix (Fisher Scientific), 0.1 µL of 100 µM of each forward and reverse primer (IDT; DNA Technology, Coralville, IA, USA), 7.3 µL of molecular grade water, and 5 µL of DNA from each sample tested.

Conventional PCR was performed in an Eppendorf Mastercycler EPgradient (Hauppauge, NY, USA) under the following conditions: 1 cycle at 95°C for 2 s, followed by 55 cycles with DNA denaturing at 94°C for 15 s, annealing at 64°C for 15 s, and extension at 72°C for 18 s. The PCR was completed by a final cycle at 72°C for 30 s. As previously described for the *Bartonella* ITS genus and *B. koehlerae*–specific PCRs, all products were analyzed by using 2% agarose gel electrophoresis and ethidium bromide under UV light, after which amplicon products were submitted to a commercial laboratory (Eton Bioscience Inc., Research Triangle Park, NC, USA) for DNA sequencing to identify the species and ITS strain type (*13**,**15**,**28**,**30*).

To check for potential contamination during processing, we simultaneously processed a noninoculated BAPGM culture flask in the biosafety hood in an identical manner for each batch of patient blood and serum samples tested. For PCR, negative controls were prepared by using 5 µL of DNA from the blood of a healthy dog. All controls remained negative throughout the course of the study.

### Statistical Analysis

Descriptive statistics were obtained for all demographic variables, self-reported clinical symptoms and concurrent conditions, previous specialist consultation, and self-reported exposures. The χ^2^ test was used to assess associations between self-reported clinical symptoms and previous specialist consultation separately with PCR results for *B. henselae*; *B. koehlerae*; and *B. vinsonii* subsp. *berkhoffii* genotypes I, II, and III. The Fisher exact test was used when expected cell value was <5. For the initial analysis, a liberal α value (α<0.10) was selected. The effect of each significant variable on the outcome variables was adjusted in separate multivariate logistic regression models controlling for age, sex, and health status. The models were repeated for different possible outcomes: PCR results for *B. henselae* or PCR results for *B. koehlerae.* Variables maintaining p<0.05 were considered significant. For some comparisons of potential interest, we were unable to estimate associations with the outcome(s) of interest because of low numbers (e.g., *B. vinsonii* subsp. *berkhoffii* genotypes I, II and III). Statistical analyses were performed by using SAS/STAT for Windows version 9.2 (SAS Institute Inc., Cary, NC, USA).

## Results

### Patient Characteristics

**Figure 1:**
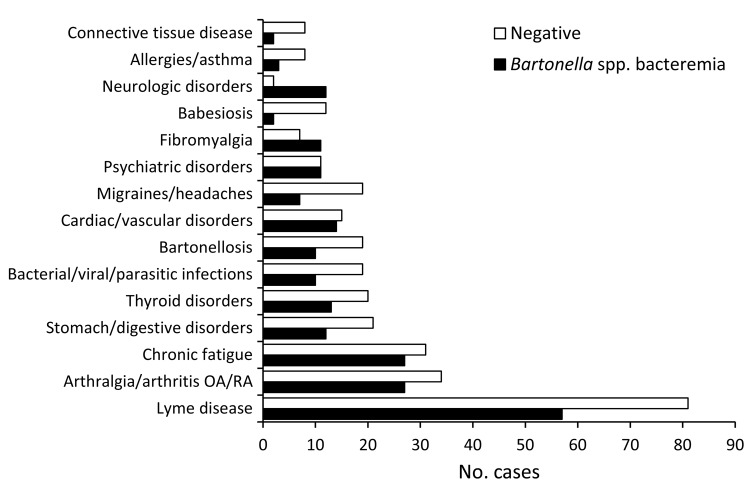
*Bartonella* spp. PCR results for the 15 most frequently reported previous diagnoses. OA, osteoarthritis; RA, rheumatoid arthritis.

The age range of the 296 patients was 3–90 years; median ages were 46 years for women and 36 years for men (Table 1). Women made up ≈70% of the study population. Most (68.2%) patients reported that they felt ill, whether chronically or infrequently, and 27.7% considered themselves to be generally healthy. The most common animal exposure reported was dog (n = 252; 85.1%), followed by cat (n = 202; 68.2%) and horse (n = 86; 29.0%). Most patients reported having been bitten or scratched by an animal (n = 202; 68.2%) or exposed to ticks (n = 229; 77.4%) and biting flies (n = 160; 54.0%). Hiking was the predominant outdoor activity reported (52.0%). Most (273 [92.2%]) patients reported having had a condition diagnosed before visiting the rheumatologist. Previously diagnosed conditions included Lyme disease (46.6%), arthralgia/arthritis or osteoarthritis/rheumatoid arthritis (20.6%), chronic fatigue (19.6%), and fibromyalgia (6.1%) (Figure 1).

**Table 1:** Characteristics and *Bartonella* spp. PCR results for 296 patients examined by a rheumatologist, Maryland–Washington, DC, USA, August 25, 2008–April 1, 2009*

Characteristic	Overall study population, no. (%)	Positive *Bartonella* sp. result by PCR, no. (%)				
		Overall positive	*B. henselae*	*B. koehlerae*	*B. vinsonii* subsp. *berkhoffii*	*Bartonella* spp.†
Total	296 (100)	122 (62.5)	40 (13.5)	54 (18.2)	10 (3.4)	29 (9.8)
Sex						
F	205 (69.3)	86 (29.0)	24 (11.7)	38 (18.5)	7 (3.4)	21 (10.3)
M	91 (30.7)	36 (12.2)	16 (17.6)	16 (17.5)	3 (3.3)	8 (8.8)
State of residence						
Maryland	148 (50.0)	58 (39.2)	20 (13.5)	27 (18.2)	5 (3.4)	13 (8.8)
Virginia	76 (25.7)	37 (48.7)	13 (17.1)	19 (25.0)	0	7 (9.2)
Pennsylvania	26 (8.8)	9 (34.6)	2 (7.7)	3 (11.5)	2 (7.7)	3 (11.5)
District of Columbia	16 (5.4)	5 (31.3)	1 (6.3)	1 (6.25)	1 (6.3)	2 (12.5)
Other	30 (10.1)	13 (43.3)	4 (13.3)	4 (13.3)	2 (6.7)	4 (13.3)
Immunofluorescence antibody results						
All *Bartonella* spp.	185 (62.5)	77 (41.6)	25 (13.5)	33 (17.8)	4 (2.1)	20 (10.8)
* B. henselae*	67 (22.6)	24 (35.8)	7 (10.3)	8 (11.7)	2 (2.9)	8 (11.7)
* B. koehlerae*	89 (30.1)	38 (42.7)	10 (11.2)	24 (26.9)	3 (3.4)	5 (5.6)
*B. vinsonii* subsp. *berkhoffii*	148 (50.0)	59 (39.8)	21 (14.1)	21 (14.1)	3 (2.0)	18 (12.1)
Self-report health assessment						
Healthy	82 (27.7)	32 (39.0)	12 (14.6)	13 (15.8)	3 (3.6)	7 (8.5)
Infrequently Ill	53 (17.9)	26 (49.1)	7 (13.2)	14 (26.4)	3 (5.6)	5 (9.4)
Chronically Ill	149 (50.3)	54 (36.2)	17 (11.4)	31 (14.1)	4 (2.7)	15 (10.1)
No response	12 (4.0)	10 (83.3)	4 (33.3)	6 (50.0)	0	2 (16.7)
Animal contact						
Yes	283 (95.6)	116 (40.9)	38 (13.4)	51 (18.0)	9 (3.2)	27 (9.5)
No	13 (4.4)	6 (46.2)	2 (15.4)	3 (23.1)	1 (7.7)	2 (15.4)
Type						
Dog	252 (85.1)	104 (41.3)	33 (13.1)	45 (17.9)	7 (2.8)	27 (10.7)
Cat	202 (68.2)	77 (38.1)	24 (11.8)	34 (16.8)	7 (3.5)	19 (9.4)
Horse	86 (29.0)	41 (47.7)	12 (13.9)	14 (16.3)	2 (2.3)	13 (15.1)
Bird	59 (19.3)	26 (44.0)	8 (13.5)	8 (13.5)	2 (3.4)	9 (15.2)
Cattle	32 (10.8)	11 (34.4)	3 (9.3)	4 (12.5)	0	4 (12.5)
Poultry	30 (10.1)	13 (43.3)	6 (20.0)	3 (10.0)	0	4 (30.7)
Swine	25 (8.5)	10 (25.0)	5 (20.0)	2 (8.0)	0	3 (12.0)
Sheep	25 (8.5)	12 (48.0)	6 (24.0)	2 (8.0)	0	4 (16.0)
Other	12 (4.0)	12 (58.3)	4 (33.3)	1 (8.3)	0	2 (16.7)
Animal bites/scratches						
Cat	154 (52.0)	64 (41.6)	21 (13.6)	27 (17.5)	6 (3.9)	14 (9.1)
Dog	118 (39.8)	52 (44.1)	18 (15.3)	22 (18.6)	2 (1.7)	13 (11.0)
Bird	12 (4.0)	10 (83.3)	3 (25.0)	4 (33.3)	2 (16.7)	3 (25.0)
Horse	14 (4.7)	9 (64.2)	2 (14.3)	3 (21.4)	1 (8.3)	3 (21.4)
Insect exposure						
Mosquitoes	256 (86.5)	106 (41.4)	37 (14.4)	46 (17.9)	8 (3.1)	24 (9.4)
Ticks	229 (77.4)	96 (41.9)	29 (12.6)	43 (18.7)	10 (4.3)	23 (10.0)
Fleas	148 (50.0)	66 (44.5)	23 (15.5)	26 (17.5)	7 (4.7)	16 (10.8)
Biting Flies	160 (54.0)	68 (42.5)	25 (15.6)	27 (16.9)	5 (3.1)	16 (10.0)
Lice	38 (12.8)	17 (44.7)	7 (18.4)	3 (7.8)	0	7 (18.4)
Spiders	5 (1.7)	4 (80.0)	1 (20.0)	2 (40.0)	0	1 (20.0)
*Sarcoptes* mite	3 (1.0)	1 (33.3)	0	0	0	1 (33.3)
Outdoor exposure						
Hiking	154 (52.0)	66 (42.9)	21 (13.6)	28 (18.2)	5 (3.3)	16 (10.4)
Wildlife rescue/rehabilitation	22 (7.4)	7 (31.8)	2 (9.1)	2 (9.1)	0	3 (14.3)
Hunting	21 (7.1)	9 (42.9)	1 (4.7)	4 (19.0)	0	4 (19.1)
Other	36 (12.2)	16 (44.4)	6 (16.7)	8 (22.2)	2 (5.6)	1 (2.8)

*Positive sample and exposure categories are not mutually exclusive (i.e., some persons had positive test results by both IFA and PCR, or could have been exposed to both cats and dogs). Median patient ages, for women and men, respectively, were as follows: overall study population, 46.0 and 36.0 y; those with positive results for overall *Bartonella*, 47.0 and 38.0 y, *B. henselae*, 44.0 and 41.0 y, *B. koehlerae*, 49.0 and 40.5 y, *B. vinsonii* subsp. *berkhoffii,* 43.0 and 64.0 y, and *Bartonella* spp., 48.0 and 24.0 y. †Positive PCR results after using *Bartonella* genus primers but unable to obtain a clean sequence to determine species.

### Serologic and BAPGM Findings

**Figure 2:**
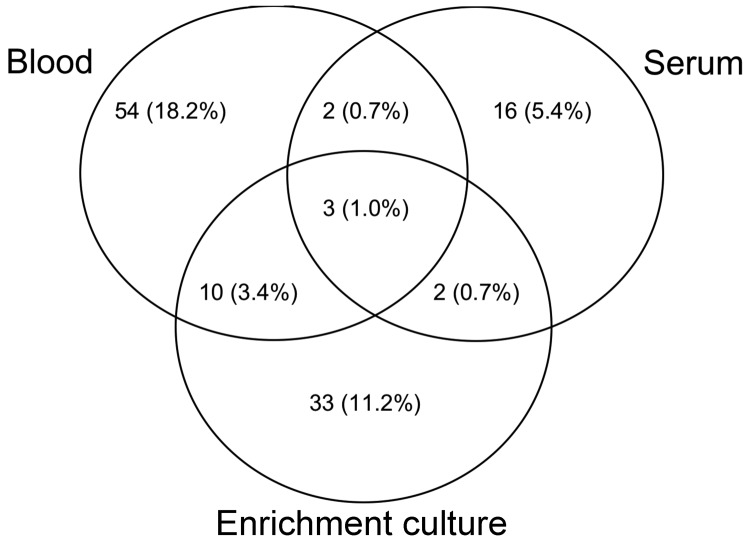
*Bartonella* PCR amplification results from blood, serum, and enrichment blood culture with the *Bartonella* α Proteobacteria growth medium. Of 296 patients, 120 had positive PCR results in 1 component. Two patients, who had positive PCR results only after enrichment culture incubation and subculture onto agar, are not included. Each circle represents *Bartonella* PCR amplification results from blood, serum, or after enrichment blood culture. Each number represents the total (%) positive for each of the 4 possibilities within each of the 3 circles. For example, only 3 (1%) patients had positive results from blood, serum, and enrichment blood culture.

Of the 296 patients, 185 (62.5%) were seroreactive to >1 *Bartonella* sp. antigens and 122 (41.1%) were infected with *B. henselae*, *B. koehlerae*, *B. vinsonii* subsp. *berkhoffii*, or *Bartonella* spp. Of the 122 patients with *Bartonella* spp. infection, PCR results were positive but DNA sequencing was unsuccessful or did not enable species identification for 29 (23.7%). After subculture, 6 isolates were obtained from 5 samples: 3 *B. henselae* isolates, 2 *B. koehlerae* isolates, and 1 *Bartonella* sp. isolate that was not fully characterized. Of the *Bartonella*-infected patients, 120 (98.4%) had a positive PCR result after DNA extraction from blood, serum, or enrichment culture (Figure 2), and 2 (1.6%) had a positive PCR result only after subculture isolation.

For *B. henselae,* 67 (22.6%) patients were seroreactive and 40 (13.5%) had positive PCR results. Of these 40 patients, only 7 (17.5%) were concurrently *B. henselae* seroreactive, whereas 33 (82.5%) patients who had a positive PCR result were not seroreactive to *B. henselae* antigens. There was no association between *B. henselae* antibodies and bacteremia (p = 0.37).

For *B. koehlerae*, 89 (30.1%) patients were seroreactive and 54 (18.2%) had positive PCR results. Of these 54 patients, 24 (44.4%) were seroreactive to *B. koehlerae* by IFA assay, whereas 29 (53.6%) were not seroreactive to *B. koehlerae* antigens. One patient with a positive *B. koehlerae* PCR result did not have a concurrent IFA test result (serum not submitted). There was an association between *B. koehlerae* seroreactivity and bacteremia (p = 0.008); seroreactive patients were more likely to be infected (odds ratio [OR] 2.25 [1.22–4.15]).

For *B. vinsonii* subsp. *berkhoffii*, 148 (50.0%) patients were seroreactive by IFA testing to at least 1 of 3 genotypes, and 10 (3.4%) had a positive PCR. Of these 10 patients, 3 were infected with genotype I, 6 were infected with genotype II, and for 1 patient the genotype could not be defined on the basis of readable DNA sequence. Seroreactivity to genotypes I, II, and III was found for 77 (26.0%), 102 (34.5%), and 82 (27.7%) patients, respectively. There was no association between *B. vinsonii* subsp. *berkhoffii* seroreactivity and bacteremia. Combined PCR and IFA assay results are summarized in Table 2. Of the patients with a positive PCR, 65% reported a prior diagnosis of Lyme disease (n = 138), bartonellosis (n = 29), or babesiosis (n = 14). Among the 138 patients with a prior diagnosis of Lyme disease, the prevalence of *Bartonella* spp. antibodies and bacteremia were 93 (67.4%) and 57 (41.3%), respectively.

**Table 2:** Test results for *Bartonella* spp. in 296 patients examined by a rheumatologist, Maryland–Washington, DC, USA, August 25, 2008–April 1, 2009*

*Bartonella* sp.	IFA–/PCR–	IFA+/PCR–	IFA+/PCR+	IFA–/PCR+
*B. henselae*	196	60	7	33
*B. koehlerae*	177	65	24	29
*B. vinsonii* subsp. *berkhoffii*	141	145	3	7
Genotype I	217	75	2	1
Genotype II	189	101	1	5
Genotype III	213	82	0	0

*IFA, indirect immunofluorescent antibody results obtained by using *B. henselae*, *B. koehlerae,* and *B. vinsonii* subsp. *berkhoffii* antigens; PCR, results obtained after PCR amplification by using *Bartonella* intergenic spacer primers, followed by attempted DNA sequencing of each amplicon.

### Factors Associated with *Bartonella* spp.

PCRs indicated the following: *B. henselae* positivity was associated (p<0.05) with blurred vision and numbness (Table 3), patients who had visited a neurologist were more likely than those who had not to be *B. henselae* positive, older median age was significantly associated with *B. koehlerae* positivity, and patients who reported paralysis were more likely to be positive for *B. vinsonii* subsp. *berkhoffii*. No associations were found for self-reported exposures (e.g., insect or animal exposure) and positive PCR for *B. henselae*, *B. koehlerae*, or *B. vinsonii* subsp. *berkhoffii*. No associations were found for *B. henselae*, *B koehlerae*, or *B vinsonii* subsp. *berkhoffii* positivity and seroreactivity.

**Table 3:** Factors associated with *Bartonella spp.* positivity by PCR, among 296 patients examined by a rheumatologist, Maryland–Washington, DC, USA, August 25, 2008–April 1, 2009*

Variable	*B. henselae,* no. (%)				*B. koehlerae,* no. (%)				*B. vinsonii subsp. berkhoffii,* no. (%)		
	Positive, n = 40	Negative, n = 256	p value†		Positive, n = 54	Negative, n = 242	p value†		Positive, n = 10	Negative, n = 286	p value†
Sex											
F	24 (60.0)	181 (70.7)	0.17		38 (70.4)	167 (69.0)	0.84		7 (70.0)	198 (69.2)	0.99
M	16 (40.0)	75 (29.3)			16 (29.6)	75 (30.1)			3 (30.0)	88 (30.7)	
Self-reported health status											
Healthy	12 (33.3)	70 (29.2)	0.77		13 (27.1)	69 (29.2)	0.11		3 (30.0)	79 (29.8)	0.59
Infrequently Ill	7 (19.4)	46 (18.5)			14 (29.2)	39 (16.5)			3 (30.0)	50 (18.3)	
Chronically Ill	17 (47.2)	132 (53.2)			21 (43.7)	128 (54.3)			4 (40.0)	145 (52.9)	
Signs or symptoms											
Fatigue	38 (95.0)	226 (88.3)	0.27		48 (88.9)	216 (89.3)	0.93		9 (90.0)	255 (89.2)	0.93
Headache	25 (62.5)	155 (60.5)	0.81		32 (59.2)	148 (61.2)	0.79		8 (80.0)	172 (60.2)	0.32
Difficulty remembering	32 (80.0)	174 (84.5)	0.12		38 (70.4)	168 (69.4)	0.89		4 (40.0)	202 (70.6)	0.07
Confusion	25 (62.5)	132 (51.5)	0.20		29 (53.7)	128 (52.9)	0.91		4 (40.0)	153 (59.6)	0.52
Disorientation	18 (45.0)	82 (32.0)	0.10		14 (25.9)	86 (35.5)	0.17		2 (20.0)	98 (34.3)	0.50
Irritability	30 (75.0)	153 (59.7)	0.06		32 (59.3)	151 (62.4)	0.66		5 (50.0)	178 (62.2)	0.51
Blurred vision	23 (57.5)	100 (39.1)	0.03		23 (42.6)	100 (41.3)	0.86		3 (30.0)	120 (41.9)	0.45
Eye pain	16 (40.0)	78 (30.5)	0.23		18 (33.3)	76 (31.4)	0.78		3 (30.0)	91 (31.8)	0.99
Sleeplessness	32 (80.0)	188 (73.4)	0.37		36 (66.7)	184 (76.0)	0.15		8 (80.0)	212 (74.1)	0.67
Insomnia	22 (55.0)	153 (59.7)	0.56		33 (61.1)	142 (58.7)	0.74		5 (50.0)	170 (59.4)	0.55
Balance problems	24 (60.0)	123 (48.0)	0.16		26 (48.2)	121 (50.0)	0.80		4 (40.0)	143 (50.0)	0.75
Tremors/shaking	17 (42.5)	92 (35.9)	0.42		17 (31.5)	92 (38.0)	0.36		5 (50.0)	104 (36.4)	0.51
Muscle weakness	28 (70.0)	161 (62.9)	0.38		36 (66.7)	153 (63.2)	0.63		8 (80.0)	181 (63.3)	0.34
Paralysis	3 (7.5)	13 (5.1)	0.52		3 (5.6)	13 (5.4)	0.95		2 (20.0)	14 (4.9)	0.04
Muscle pain	31 (77.5)	176 (68.7)	0.26		36 (66.7)	171 (70.6)	0.56		6 (60.0)	201 (70.3)	0.49
Numbness	28 (70.0)	128 (50.0)	0.01		25 (46.3)	131 (54.1)	0.29		7 (70.0)	149 (52.1)	0.34
Joint pain	31 (77.5)	199 (77.3)	0.97		41 (75.9)	189 (78.1)	0.73		10 (100)	220 (76.9)	0.12
Chronic fatigue	27 (67.5)	180 (70.3)	0.71		37 (68.5)	170 (70.3)	0.80		7 (70.0)	200 (69.9)	0.99
Bowel/bladder dysfunction	17 (42.5)	95 (37.1)	0.51		19 (35.2)	93 (38.4)	0.66		5 (50.0)	107 (37.4)	0.41
Shortness of breath	19 (47.5)	98 (38.3)	0.26		21 (38.9)	96 (39.7)	0.91		3 (30.0)	114 (39.8)	0.74
Poor appetite	8 (20.0)	75 (29.3)	0.22		12 (22.2)	71 (29.3)	0.29		1 (10.0)	82 (28.6)	0.29
Weight loss	7 (17.5)	52 (20.3)	0.67		6 (11.1)	53 (21.9)	0.07		1 (10.0)	58 (20.3)	0.69
Depression	20 (50.0)	126 (49.2)	0.92		28 (51.9)	118 (48.7)	0.68		4 (40.0)	142 (49.6)	0.75
Syncope	4 (10.0)	41 (16.0)	0.32		8 (14.8)	37 (5.3)	0.93		2 (20.0)	43 (15.0)	0.66
Consultation with neurologist	23 (57.5)	87 (33.9)	<0.01		22 (40.7)	88 (36.4)	0.56		3 (30.0)	107 (37.4)	0.63
Consultation with infectious disease physician	16 (40.0)	104 (40.6)	0.94		29 (53.7)	91 (37.6)	0.03		4 (40.0)	116 (40.7)	0.97

†Median ages, compared by using Wilcoxon rank-sum test, for positive and negative results, respectively, were *B. henselae*, 42.5, 44.0, p = 0.43; *B. koehlarae*, 48.0, 43.0, p = 0.03; and *B. vinsonii* subsp. *berkhoffii*, 46.5, 44.0, p = 0.64. *Results of χ^2^ analysis (Fisher exact test used when expected cell value <5).

### Logistic Regression Analysis

To identify factors associated with PCR positivity for *B. henselae* or *B. koehlerae*, we adjusted the models for 3 biological confounders: age, sex, and health status (Table 4). We identified the following factors as associated with *B. henselae–*positive PCR result: blurred vision (adjusted OR [aOR] 2.37, 95% CI 1.13–4.98), numbness (aOR 2.74, 95% CI 1.26–5.96), and previous consultation with a neurologist (aOR 2.76, 95% CI 1.33–5.73). No self-reported symptoms were significantly associated with PCR positivity for *B. koehlerae*. However, patients who had visited an infectious disease physician were more likely to have a*. B. koehlerae*–positive PCR result (aOR 1.98, 95% CI 1.05–3.75).

**Table 4:** Factors associated with positive PCR result for *Bartonella henselae* and *B*. *koehlerae among* 296 patients examined by a rheumatologist, Maryland–Washington, DC, USA, August 25, 2008–April 1, 2009*

Variable	Positive vs. negative result, adjusted odds ratio (95% CI)	
	*B. henselae*	*B. koehlerae*
Blurred vision	2.37 (1.13–4.98), p = 0.03	NS
Numbness	2.74 (1.26–5.96), p = 0.01	NS
Consultation with infectious disease physician	NS	1.98 (1.05–3.75), p = 0.04
Consultation with neurologist	2.76 (1.33–5.73), p<0.01	NS

*Results of logistic regression analysis. Variables adjusted for age, sex, and duration of illness. NS, not significant.

## Discussion

We identified unexpectedly high serologic and molecular prevalence for *B. henselae*, *B. koehlerae*, and *B. vinsonii* subsp. *berkhoffii* in patients who had been examined by a rheumatologist, of whom more than half reported a prior diagnosis of Lyme disease, bartonellosis, or babesiosis. However, the diagnostic criterion upon which these infections were based was not available for review because all prior diagnoses were self-reported. Overall, 185 (62.5%) of 296 patients had antibodies to *B. henselae*, *B. koehlerae,* or *B. vinsonii* subsp. *berkhoffii*, and 122 (41.1%) were positive for *Bartonella* spp. according to PCR. In most instances, DNA sequencing of the amplified product facilitated identification of the infecting species. The prevalence of antibodies against *Bartonella* spp. (93 [67.4%]) and bacteremia [57 [1.3%]) among 138 patients with a prior diagnosis of Lyme disease did not differ from that of the overall study population. Because our analysis was restricted to patients selected by a rheumatologist practicing in a Lyme disease–endemic region, extrapolations to other regions or other rheumatology practices might not be applicable. Also, because the survey was self-administered, objective confirmation of symptoms, conditions, and diagnoses was not always possible; therefore, responses might have been subject to respondent bias. Similarly, because responses associated with symptoms, conditions, and exposures might have occurred over a protracted time, survey responses might also be subject to recall bias.

Despite these study limitations, *B. henselae* infections seemed to be more common in patients who reported blurred vision, numbness in the extremities, and previous consultation with a neurologist before referral to the rheumatologist. In a case series of 14 patients, the following were reported by 50% of patients infected with a *Bartonella* species, specifically *B. henselae*, *B. vinsonii* subsp. *berkhoffii*, or both: memory loss, numbness or a loss of sensation, balance problems, and headaches (*10*). Another 6 *B. henselae*–bacteremic patients reported seizures, ataxia, memory loss, and/or tremors; 1 of these patients was co-infected with *B. vinsonii* subsp. *berkhoffii*, and another was positive for *B. henselae* by PCR after enrichment of cerebrospinal fluid in BAPGM (*23*). An enrichment culture approach also identified an association between intravascular infection with *B*. *vinsonii* subsp. *berkhoffii* genotype II and *B. henselae* and neurologic symptoms in a veterinarian and his daughter (*12*). Symptoms in the father included progressive weight loss, muscle weakness, and lack of coordination; symptoms in the daughter were headaches, muscle pain, and insomnia. For each patient, after repeated courses of antimicrobial drugs, blood cultures became negative, antibody titers decreased to nondetectable levels, and all neurologic symptoms resolved.

Although no symptoms were statistically associated with *B. koehlerae* infection, patients infected with *B. koehlerae* were more likely to have previously consulted an infectious disease physician. Of the 54 *B. koehlerae* patients with a positive PCR result, 54% reported a prior diagnosis of Lyme disease (n = 25), bartonellosis (n = 3), or babesiosis (n = 1). Fatigue, insomnia, memory loss, and joint and muscle pain were frequent complaints among those with a positive PCR result for *B. koehlerae*, but these symptoms did not differ in frequency from those in patients with negative PCR. Similar symptoms were previously reported in a small case series involving *B. koehlerae*–bacteremic patients (*13*). Peripheral visual deficits, sensory loss, and hallucinations resolved in a young woman after antimicrobial drug treatment for *B. koehlerae* infection (*30*). Because of the small number of patients with positive PCR results for *B. vinsonii* subsp. *berkhoffii*, we restricted the multivariate analysis to those with positive results for *B. henselae* and *B. koehlerae*. Because limited sample size affected our ability to conduct multivariate analysis to control for potential confounders for *B. vinsonii* subsp. *berkhoffii* positivity, the χ^2^ associations with *B. vinsonii* subsp. *berkhoffii* positivity should be interpreted with caution.

Although the pathogenic relevance of the high *Bartonella* spp. seroprevalence and bacteremia in this patient population are unclear, these results justify additional prospective studies involving more narrowly defined patient and control populations. Of the 92 patients infected with *B. koehlerae, B. henselae,* or *B. vinsonii* subsp. *berkhoffi*, 69 (75%) had at least 1 discordant IFA assay result for *Bartonella* spp. antigen seroreactivity and only 34 (30.6%) had a concordant species-specific PCR and IFA result. Also, consistent with previous study findings (*15*), the PCRs depicted in Figure 2 illustrate an increased likelihood of positivity if blood, serum, and enrichment blood cultures are independently tested. According to these and previous results (*7**,**18**,**31**,**32*), a subset of *Bartonella* spp.–bacteremic patients could be anergic and might not produce a detectable IFA response, or alternatively, the substantial antigenic variation among various *Bartonella* strains might result in false-negative IFA assay results for some patients. In a study on *Bartonella* serology conducted by the Centers for Disease Control and Prevention, IFA cross-reactivity among *Bartonella* species occurred in 94% of patients with suspected cat-scratch disease (*33*). Despite the lack of concordance between serologic results and BAPGM enrichment PCR results, most (185 [62.5%]) patients in our study were seroreactive to *Bartonella* spp., suggesting prior exposure to >1 *Bartonella* spp. Because serologic cross-reactivity to *Chlamydia* spp. and *Coxiella burnettii* antigens has been reported, exposure to these or other organisms might have contributed to the high seroprevalence. In a previous study involving 32 healthy volunteers and patients at high risk for *Bartonella* spp. bacteremia, seroprevalence rates for *B. henselae*, *B. koehlerae* and *B. vinsonii* subsp. *berkhoffii* genotypes I and II were 3.1%, 0%, 0,%, and 50%, respectively, for the healthy population compared with 15.6%, 9.2%, 19.8%, and 28.1%, respectively, for the high-risk population (*15*). Although in that study and the study reported here, the same test antigens and identical IFA assays were used and the same research technologist interpreted the results, the overall seroprevalence in the study reported here was higher than that among high-risk patients with extensive arthropod or animal contact (49.5%) and differed substantially from serologic results from healthy volunteers (*15*). However, in the study reported here, a large portion of the population (34.5%) was also seroreactive to *B. vinsonii berkhoffii* genotype II. Immunophenotypic properties giving rise to seroreactivity to this particular antigen among healthy control and patient populations have not been clarified but could be related to polyclonal B-cell activation, commonly found in patients with rheumatologic or chronic inflammatory diseases.

It is becoming increasingly clear that no single diagnostic strategy will confirm infection with a *Bartonella* sp. in immunocompetent patients. Before the current study, we primarily used BAPGM enrichment blood cultures and PCR to test symptomatic veterinarians, veterinary technicians, and wildlife biologists, who seem to be at occupational risk for *Bartonella* sp. bacteremia because of animal contact and frequent arthropod exposure (*10**–**15**,**23*). Cats are the primary reservoir hosts for *B. henselae* and *B. koehlerae,* whereas canids, including dogs, coyotes and foxes, are the primary reservoir hosts for *B. vinsonii* subsp. *berkhoffii (**4**,**6**,**29**,**34**)*. Although infrequent when compared with cat transmission of *B. henselae* resulting in classical cat-scratch disease, dogs have been implicated in the transmission of *B. vinsonii* subsp. *berkhoffii* and *B. henselae* to humans (*35**,**36*). The predominant symptoms reported among occupationally at-risk patient populations have included severe fatigue, neurologic and neurocognitive abnormalities, arthralgia, and myalgia (*10**–**13**,**23*). In the study reported here, dog (85%) and cat (68%) contact were reported by most respondents; however, no associations were found between infection with a *Bartonella* sp. and contact with a specific animal. Similarly, exposure to mosquitoes, ticks, fleas, and biting flies were all reported by >50% of the study population. The results of this study support documentation of *Bartonella* spp. bacteremia in patients seen by a rheumatologist in a Lyme disease–endemic area and provides the basis for future studies to ascertain the prevalence of *Bartonella* spp. in patients with rheumatic and neurologic symptoms.

## References

[1] Dehio C. Interactions of *Bartonella henselae* with vascular endothelial cells. Curr Opin Microbiol. 1999;2:78–82. 10.1016/S1369-5274(99)80013-7 10047560

[2] Kordick DL, Breitschwerdt EB. Persistent infection of pets within a household with three *Bartonella* species. Emerg Infect Dis. 1998;4:325–8. 10.3201/eid0402.980225 9621208 PMC2640143

[3] Kosoy MY, Regnery RL, Tzianabos T, Marston EL, Jones DC, Green D, Distribution, diversity, and host specificity of *Bartonella* in rodents from the southeastern United States. Am J Trop Med Hyg. 1997;57:578–88. 9392599 10.4269/ajtmh.1997.57.578

[4] Abbott RC, Chomel BB, Kasten RW, Floyd-Hawkins KA, Kikuchi Y, Koehler JE, Experimental and natural infection with *Bartonella henselae* in domestic cats. Comp Immunol Microbiol Infect Dis. 1997;20:41–51. 10.1016/S0147-9571(96)00025-2 9023040

[5] Boulouis HJ, Chang CC, Henn JB, Kasten RW, Chomel BB. Factors associated with the rapid emergence of zoonotic *Bartonella* infections. Vet Res. 2005;36:383–410. 10.1051/vetres:2005009 15845231

[6] Chomel BB, Boulouis HJ, Maruyama S, Breitschwerdt EB. *Bartonella* spp. in pets and effect on human health. Emerg Infect Dis. 2006;12:389–94. 10.3201/eid1203.050931 16704774 PMC3291446

[7] Jendro MC, Weber G, Brabant T, Zeidler H, Wollenhaupt J. Reactive arthritis after cat bite: a rare manifestation of cat scratch disease—case report and overview [in German]. Z Rheumatol. 1998;57:159–63. 10.1007/s003930050074 9702836

[8] Chomel BB, Kasten RW, Sykes JE, Boulouis HJ, Breitschwerdt EB. Clinical impact of persistent *Bartonella* bacteremia in humans and animals. Ann N Y Acad Sci. 2003;990:267–78. 10.1111/j.1749-6632.2003.tb07376.x 12860639

[9] Rolain JM, Brouqui P, Koehler JE, Maguina C, Dolan MJ, Raoult D. Recommendations for treatment of human infections caused by *Bartonella* species. Antimicrob Agents Chemother. 2004;48:1921–33. 10.1128/AAC.48.6.1921-1933.2004 15155180 PMC415619

[10] Breitschwerdt EB, Maggi RG, Duncan AW, Nicholson WL, Hegarty BC, Woods CW. *Bartonella* species in blood of immunocompetent persons with animal and arthropod contact. Emerg Infect Dis. 2007;13:938–41. 17553243 10.3201/eid1306.061337 PMC2792845

[11] Breitschwerdt EB, Maggi RG, Farmer P, Mascarelli PE. Molecular evidence of perinatal transmission of *Bartonella vinsonii* subsp. *berkhoffii* and *Bartonella henselae* to a child. J Clin Microbiol. 2010;48:2289–93. 10.1128/JCM.00326-10 20392912 PMC2884525

[12] Breitschwerdt EB, Maggi RG, Lantos PM, Woods CW, Hegarty BC, Bradley JM. *Bartonella vinsonii* subsp. *berkhoffii* and *Bartonella henselae* bacteremia in a father and daughter with neurological disease. Parasites & Vectors. 2010;3:29. 10.1186/1756-3305-3-29 20377863 PMC2859367

[13] Breitschwerdt EB, Maggi RG, Mozayeni BR, Hegarty BC, Bradley JM, Mascarelli PE. PCR amplification of *Bartonella koehlerae* from human blood and enrichment blood cultures. Parasites & Vectors. 2010;3:76. 10.1186/1756-3305-3-76 20735840 PMC2936392

[14] Breitschwerdt EB, Maggi RG, Varanat M, Linder KE, Weinberg G. Isolation of *Bartonella vinsonii* subsp. *berkhoffii* genotype II from a boy with epithelioid hemangioendothelioma and a dog with hemangiopericytoma. J Clin Microbiol. 2009;47:1957–60. 10.1128/JCM.00069-09 19369441 PMC2691088

[15] Maggi RG, Mascarelli PE, Pultorak EL, Hegarty BC, Bradley JM, Mozayeni BR, *Bartonella* spp. bacteremia in high-risk immunocompetent patient. Diagn Microbiol Infect Dis. 2011;71:430–7. 10.1016/j.diagmicrobio.2011.09.001 21996096

[16] Al-Matar MJ, Petty RE, Cabral DA, Tucker LB, Peyvandi B, Prendiville J, Rheumatic manifestations of *Bartonella* infection in 2 children. J Rheumatol. 2002;29:184–6. 11824958

[17] Giladi M, Maman E, Paran D, Bickels J, Comaneshter D, Avidor B, Cat-scratch disease–associated arthropathy. Arthritis Rheum. 2005;52:3611–7. 10.1002/art.21411 16255053

[18] Hayem F, Chacar S, Hayem G. *Bartonella henselae* infection mimicking systemic onset juvenile chronic arthritis in a 2½-year-old girl. J Rheumatol. 1996;23:1263–5. 8823702

[19] Maman E, Bickels J, Ephros M, Paran D, Comaneshter D, Metzkor-Cotter E, Musculoskeletal manifestations of cat scratch disease. Clin Infect Dis. 2007;45:1535–40. 10.1086/523587 18190312

[20] Tsukahara M, Tsuneoka H, Tateishi H, Fujita K, Uchida M. *Bartonella* infection associated with systemic juvenile rheumatoid arthritis. Clin Infect Dis. 2001;32:E22–3. 10.1086/317532 11112671

[21] Dillon B, Cagney M, Manolios N, Iredell JR. Failure to detect *Bartonella henselae* infection in synovial fluid from sufferers of chronic arthritis. Rheumatol Int. 2000;19:219–22. 10.1007/PL00006854 11063291

[22] Breitschwerdt EB, Suksawat J, Chomel B, Hegarty BC. The immunologic response of dogs to *Bartonella vinsonii* subspecies *berkhoffii* antigens: as assessed by Western immunoblot analysis. J Vet Diagn Invest. 2003;15:349–54. 10.1177/104063870301500408 12918816

[23] Breitschwerdt EB, Maggi RG, Nicholson WL, Cherry NA, Woods CW. *Bartonella* sp. bacteremia in patients with neurological and neurocognitive dysfunction. J Clin Microbiol. 2008;46:2856–61. 10.1128/JCM.00832-08 18632903 PMC2546763

[24] Diniz PP, Maggi RG, Schwartz DS, Cadenas MB, Bradley JM, Hegarty B, Canine bartonellosis: serological and molecular prevalence in Brazil and evidence of co-infection with *Bartonella henselae* and *Bartonella vinsonii* subsp. *berkhoffii.* Vet Res. 2007;38:697–710. 10.1051/vetres:2007023 17583666

[25] Duncan AW, Maggi RG, Breitschwerdt EB. A combined approach for the enhanced detection and isolation of *Bartonella* species in dog blood samples: pre-enrichment liquid culture followed by PCR and subculture onto agar plates. J Microbiol Methods. 2007;69:273–81. 10.1016/j.mimet.2007.01.010 17346836

[26] Maggi RG, Duncan AW, Breitschwerdt EB. Novel chemically modified liquid medium that will support the growth of seven *Bartonella* species. J Clin Microbiol. 2005;43:2651–5. 10.1128/JCM.43.6.2651-2655.2005 15956379 PMC1151927

[27] Cadenas MB, Bradley J, Maggi RG, Takara M, Hegarty BC, Breitschwerdt EB. Molecular characterization of *Bartonella vinsonii* subsp. *berkhoffii* genotype III. J Clin Microbiol. 2008;46:1858–60. 10.1128/JCM.02456-07 18367567 PMC2395075

[28] Maggi RG, Breitschwerdt EB. Potential limitations of the 16S–23S rRNA intergenic region for molecular detection of *Bartonella* species. J Clin Microbiol. 2005;43:1171–6. 10.1128/JCM.43.3.1171-1176.2005 15750079 PMC1081238

[29] Maggi RG, Chomel B, Hegarty BC, Henn J, Breitschwerdt EB. A *Bartonella vinsonii berkhoffii* typing scheme based upon 16S–23S ITS and Pap31 sequences from dog, coyote, gray fox, and human isolates. Mol Cell Probes. 2006;20:128–34. 10.1016/j.mcp.2005.11.002 16460911

[30] Breitschwerdt EB, Mascarelli PE, Schweickert LA, Maggi RG, Hegarty BC, Bradley JM, Hallucinations, sensory neuropathy, and peripheral visual deficits in a young woman infected with *Bartonella koehlerae.* J Clin Microbiol. 2011;49:3415–7. 10.1128/JCM.00833-11 21734026 PMC3165616

[31] Diniz PP, Wood M, Maggi RG, Sontakke S, Stepnik M, Breitschwerdt EB. Co-isolation of *Bartonella henselae* and *Bartonella vinsonii* subsp. *berkhoffii* from blood, joint and subcutaneous seroma fluids from two naturally infected dogs. Vet Microbiol. 2009;138:368–72. 10.1016/j.vetmic.2009.01.038 19560291

[32] Jones SL, Maggi R, Shuler J, Alward A, Breitschwerdt EB. Detection of *Bartonella henselae* in the blood of 2 adult horses. J Vet Intern Med. 2008;22:495–8. 10.1111/j.1939-1676.2008.0043.x 18346149

[33] Dalton MJ, Robinson LE, Cooper J, Regnery RL, Olson JG, Childs JE. Use of *Bartonella* antigens for serologic diagnosis of cat-scratch disease at a national referral center. Arch Intern Med. 1995;155:1670–6. 10.1001/archinte.1995.00430150164017 7542443

[34] Breitschwerdt EB, Kordick DL. *Bartonella* infection in animals: carriership, reservoir potential, pathogenicity, and zoonotic potential for human infection. Clin Microbiol Rev. 2000;13:428–38. 10.1128/CMR.13.3.428-438.2000 10885985 PMC88941

[35] Keret D, Giladi M, Kletter Y, Wientroub S. Cat-scratch disease osteomyelitis from a dog scratch. J Bone Joint Surg Br. 1998;80:766–7. 10.1302/0301-620X.80B5.8823 9768882

[36] Tsukahara M, Tsuneoka H, Iino H, Ohno K, Murano I. *Bartonella henselae* infection from a dog. Lancet. 1998;352:1682. 10.1016/S0140-6736(05)61455-9 9853451

